# Usefulness of High-resolution Computed Tomography for Macrolide Therapy of Idiopathic Bronchiectasis

**DOI:** 10.2174/18743064-v17-230822-2022-27

**Published:** 2023-08-29

**Authors:** Zenya Saito, Masahiro Yoshida, Shota Uchiyama, Saiko Nishioka, Kentaro Tamura, Nobumasa Tamura

**Affiliations:** 1 Division of Respiratory Diseases, Department of Internal Medicine, Atsugi City Hospital, Kanagawa , Japan

**Keywords:** Bronchiectasis, Exacerbation, Respiratory disease, High-resolution computed tomography, Macrolide, Assessment

## Abstract

**Background:**

High-resolution computed tomography (HRCT) correlates with clinical symptoms, respiratory function, and quality of life in bronchiectasis.

**Objective:**

We aimed to investigate the relationship between macrolide and acute exacerbation (AE) in idiopathic bronchiectasis classified by the Bronchiectasis Radiologically Indexed CT Score (BRICS).

**Methods:**

We retrospectively reviewed the medical records of patients diagnosed with idiopathic bronchiectasis between April 2014 and December 2020 at a single hospital. Overall, 115 patients with idiopathic bronchiectasis were selected and divided into three groups, according to the BRICS. Each group was divided into subgroups with and without macrolide therapy, and the number of patients with AE in each group was retrospectively compared.

**Results:**

About 45, 48, and 22 patients were included in the mild, moderate, and severe groups, respectively. In the mild group, the subgroup with macrolide therapy had significantly fewer patients with single AE than those without macrolide ( *P* = 0.029). There was no significant difference in the moderate and severe groups ( *P* = 1.00 and 0.64, respectively). In the multiple AE, the subgroup with macrolide therapy had significantly fewer patients than those without macrolide therapy in the mild, moderate, and severe groups ( *P* = 0.024, 0.029, and 0.026, respectively).

**Conclusion:**

HRCT severity assessment might be useful in predicting treatment efficacy in patients with idiopathic bronchiectasis without previous AEs. Further large-scale clinical trials are required on the usefulness of HRCT in the future.

## 1 INTRODUCTION

Bronchiectasis causes chronic lower respiratory tract infections due to persistent inflammation [ 1- 3]. However, secondary bronchiectasis often results from some other inflammatory diseases or infections, such as rheumatoid arthritis, ulcerative colitis, nontuberculous mycobacteriosis, and diffuse panbronchiolitis (DPB), idiopathic bronchiectasis [ 4, 5], accounting for about 50% of all cases [ 6- 8]. Long-term, low-dose macrolide therapy, originally performed for DPB by Kudo *et al.* [ 9], was applied to bronchiectasis and was reportedly effective in several randomized controlled trials [ 10- 12]. They indicated various anti-inflammatory and anti-microbial effects of macrolide therapy, including prevention of inflammatory cell migration, decrease of active oxygen production, *Pseudomonas aeruginosa* biofilm formation, and excessive airway mucus. In 2017, the European Respiratory Society guidelines recommended long-term antibiotic treatment for adults with bronchiectasis with three or more acute exacerbations (AEs) per year without *P. aeruginosa* infection [ 13]. In the latest meta-analysis, however, macrolide antibiotics reduced the frequency of AE in patients with *P. aeruginosa* and patients with one to two exacerbations per year. Furthermore, this treatment improved the time to first exacerbation and quality of life measured by the St. George’s Respiratory Questionnaire [ 14]. Therefore, in contrast to current guidelines, macrolides may also be effective in patients who are not indicated for this treatment.

High-resolution computed tomography (HRCT) is the gold standard for diagnosing bronchiectasis. In 1991, Bhalla *et al*. suggested an HRCT scoring system that used nine radiological categories [ 15]. A recent study developed the bronchiectasis radiologically indexed CT score (BRICS) using bronchial dilatation parameters and the number of bronchopulmonary segments with emphysema. BRICS demonstrated that radiological appearances alone could reflect the severity of the disease [ 16].

There is no consensus regarding indications of using long-term, low-dose macrolide therapy in bronchiectasis. Moreover, no studies have investigated the relationship between the effect of macrolide therapy and the severity assessment performed by HRCT in idiopathic bronchiectasis. In contrast, HRCT correlates with clinical symptoms, respiratory function, and quality of life in patients with bronchiectasis [ 17]. In short, unlike patients with mild bronchiectasis, we assume that the therapeutic effects in patients with severe bronchiectasis, for whom a wide range of airways is irreversibly destroyed, are limited. We classified patients with idiopathic bronchiectasis by severity based on the BRICS system and compared the effects of long-term, low-dose macrolide therapy with AEs.

## 2 MATERIALS AND METHODS

### 2.1 Study Population and Selection Criteria

We retrospectively reviewed the medical records of patients diagnosed with idiopathic bronchiectasis between April 2014 and December 2020 at a single hospital. Patients were enrolled through a hospital database, and we extracted clinical data from their electronic medical records.

The inclusion criteria were as follows: (1) patients who had bronchial dilatation, visibility of peripheral airways, bronchial wall thickening, and small airway abnormality on HRCT confirmed by multiple radiologists and pulmonologists; (2) patients who had regular outpatient clinic visits for at least 2 years; and (3) patients who had continuous macrolide therapy for 2 years regardless of the therapeutic effect. In contrast, the exclusion criteria were as follows: (1) patients with secondary bronchiectasis due to rheumatoid arthritis, ulcerative colitis, DPB, nontuberculous mycobacteriosis, and cystic fibrosis were ruled out by clinical symptoms, serological test, bacteriological examination, and/or endoscopy; (2) patients who were under macrolide therapy for other diseases; (3) patients who discontinued macrolide therapy due to dropouts or adverse events; (4) in cases where disagreements between pulmonologists and radiologists regarding patients’ HRCT findings were not resolved by consensus; (5) patients with interstitial pneumonia and malignant tumors; and (6) patients who had AE in the past. The Atsugi City Hospital Ethics Committee approved this study. All patient records were anonymized before analysis, and all patients obtained informed consent (approval number, R2-02). This committee approved the verbal consent procedure. We used an opt-out method and disclosed this to the patients.

### 2.2 Data Analysis and Comparison Factors

**Fig. (1):**
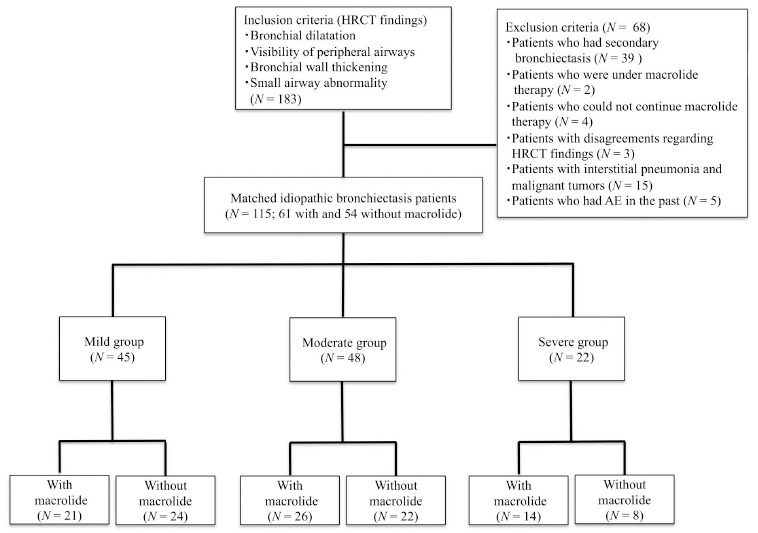
Patient selection flow. Legend: Of the 115 eligible patients diagnosed with idiopathic bronchiectasis, 45, 48, and 22 were included in the mild, moderate, and severe groups, respectively. Of the 45 patients in the mild group, 21 and 24 were assigned to the with and without macrolide subgroups, respectively. Of the 48 patients in the moderate group, 26 and 22 were assigned to the with and without macrolide subgroups, respectively. Of the 22 patients in the severe group, 14 and 8 were assigned to the with and without macrolide subgroups, respectively.

First, we divided all patients who met the study criteria into three groups according to the severity assessment score by BRICS (Table **1**) (score 1 indicates mild disease, score 2–3 indicates moderate disease, and score >3 indicates severe disease). Then, each group was divided into subgroups with and without long-term, low-dose macrolide therapy (Fig. **1**). We compared the clinical characteristics and the number of cases of single and multiple AE between the groups with and without macrolide therapy, and the same comparison was retrospectively performed between the subgroups with and without macrolide therapy in mild, moderate, and severe groups. Sex, age, body mass index, smoking status, clinical symptoms, forced vital capacity, and forced expiratory volume in the first second of expiration percent predicted, bacterial colonization, treatment, and comorbidities were the variables set for comparison from the electronic medical records at the start of observation.

### 2.3 Review of Radiology

**Fig. (2):**
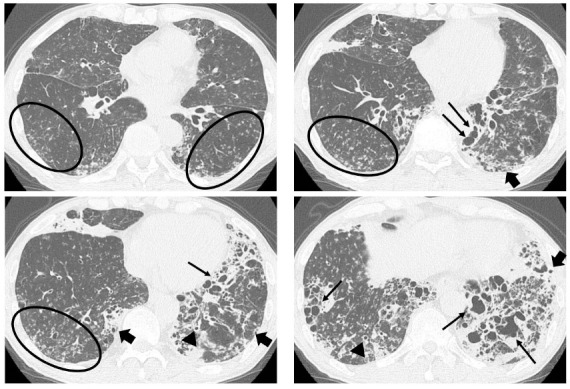
HRCT in a case of severe bronchiectasis. Legend: Bronchial dilatation (thin arrow), visibility of peripheral airway (thick arrow), bronchial wall thickening (triangle), and small airway abnormality (circle) were found in both lungs.

All patients underwent HRCT, and the radiologists’ reports were available. Abnormal findings were described as bronchial dilatation, bronchial wall thickening, visibility of peripheral airway, and small airway abnormality by multiple pulmonologists and radiologists (Fig. **2**). Bronchial dilatation was defined when the ratio between the diameter of the bronchus and the pulmonary artery running parallel (broncho-arterial ratio) was more than one. The visibility of the peripheral airway was optimal when the bronchus was within 1cm of the pleura. Small airway abnormalities included tree-in-bud appearance, centrilobular granular shadows, and mosaic attenuation. HRCT evaluated all patients within three months from the start of the observation period.

### 2.4 Definition of Long-term, Low-dose Macrolide Therapy, and AE

Low-dose macrolide therapy in all patients consisted of 400–600 mg erythromycin or 200–400 mg clarithromycin. All patients who underwent macrolide therapy received macrolide antibiotics for at least 2 years, except for dropout or discontinuation cases due to side effects.

AE presenting with acute deterioration (usually over several days) with worsening local symptoms (cough, increased sputum volume or change of viscosity, increased sputum purulence with or without increasing wheeze, breathlessness, and hemoptysis) and/or systemic upset. For ancillary diagnosis, the elevation of serum CRP, detection of bacteria in sputum culture test, shadows suggestive of pneumonia by radiologic image, and improvement of abnormal shadow by antibiotic treatment were confirmed.

### 2.5 Severity Classification on HRCT

In 1991, the Bhalla score was calculated by scoring nine radiological categories (severity of bronchiectasis, peribronchial thickening, number of bronchopulmonary segments involved, the extent of mucus plugging, sacculations or abscesses, generations of bronchial divisions involved, number of bullae, number of bronchopulmonary segments with emphysema, and collapse of consolidation), which were then added up to the total score. In this score, bronchial dilatation and the number of bronchopulmonary segments with emphysema were the two components that were significantly associated with the disease severity markers. BRICS was developed with these two parameters, and it could be used as an adjunct to clinical parameters to predict disease severity in patients with idiopathic and post-infective bronchiectasis. The score ranges from 0 to 5, and a higher score indicates increasing disease severity (1 indicates mild disease, 2–3 indicates moderate disease, and >3 indicates severe disease) (Table **1**). This study classified all patients into three groups (mild, moderate, and severe groups) using this scoring system.

### 2.6 Statistical Analysis

The average, standard deviation, median, 25 ^th^, and 75 ^th^ percentile points, and range were calculated for continuous variables. Frequency and ratio were calculated for discrete variables. For intergroup comparisons, Fisher’s exact test was used for discrete variables and the Bonferroni correction for pairwise comparisons. All groups were compared using analysis of variance and pair comparisons by *t*-test in the case of the parametric method for comparison of continuous variables. All groups were compared using the Kruskal–Wallis test for the nonparametric method, and pair comparisons were made using the Mann–Whitney *U* test. A logistic regression analysis investigated the relationship between macrolide therapy and AE, and a value of *P* < 0.05 indicated statistical significance. Statistical analyses were performed using SPSS software, version 23.0 (IBM Japan, Ltd., Tokyo, Japan).

## 3 RESULTS

Of the 115 patients with idiopathic bronchiectasis, 61 (53%) and 54 (47%) were included in the groups with and without macrolide therapy. There were no significant differences in clinical characteristics, as presented in Table **2**.

Additionally, as shown in Table **3**, there was no significant difference in a single AE between the groups with and without macrolide therapy [Odds ratio (OR)= 0.65; 95% confidence interval (CI): 0.72–3.22; *P* = 0.34]; however, patients in the macrolide group had significantly lower multiple AEs compared to the group without macrolide therapy (OR = 0.18; 95% CI: 0.07–0.47; *P* = 0.0003).

Regarding severity, 45 (mild group; 21 and 24 for with and without macrolide therapy, respectively), 48 (moderate group; 26 and 22 for with and without macrolide therapy, respectively), and 22 (severe group; 14 and 8 for with and without macrolide therapy, respectively) were assigned to each subgroup. As shown in Table **4**, no significant difference in the clinical characteristics in the mild, moderate, and severe groups was observed. However, the complication rate of respiratory symptoms, severe respiratory dysfunction, and bacterial infections, including *P. aeruginosa*, were higher in proportion to the severity. Regarding the incidence of a single AE, the subgroup with macrolide therapy had significantly fewer patients with AEs than the mild group without macrolide therapy (OR = 0.19; 95% CI: 0.05–0.77; *P* = 0.029), as shown in Table **5**. In contrast, no significant difference was found between subgroups in the moderate and severe groups, respectively (OR = 1.02 and 2.25; 95% CI: 0.32–3.21 and 0.33–15.3; *P* = 1.00 and 0.64, respectively), as shown in Table **5**. On the other hand, the subgroup with macrolide therapy had significantly fewer patients with multiple AEs regardless of severity compared to the group without macrolide therapy (mild group; OR = 0.10, moderate; 0.21, severe; 0.09; 95% CI: 0.01–0.88, 0.05–0.84, and 0.01–0.70; *P* = 0.024, 0.029, and 0.026, respectively).

## 4 DISCUSSION

Bronchiectasis is a disease that causes repeated chronic respiratory tract infections, leading to gradual but irreversible lung distortion. The pathogenesis of this condition has been previously reported as follows. First, bacterial colonization weakens the bronchi, resulting in the induction of an excessive immune response. Second, persistent bronchial inflammation destroys bronchial elastic fibers, smooth muscle, and cartilage and promotes excessive mucus secretion, ciliary movement reduction, and airway macrophage dysfunction. This leads to lung distortion and bacterial colonization, which eventually facilitates a destructive inflammation cycle [ 3, 18]. The effects of genetic predisposition and environmental factors have both been considered in idiopathic bronchiectasis; however, specific details remain unclear [ 2, 3, 18]. Long-term, low-dose macrolide therapy has been proven to be an effective treatment for bronchiectasis [ 10- 12]. Current European guidelines suggest that patients with three or more AE per year without *P. aeruginosa* infection should receive macrolide therapy [ 13]. In addition, the BLESS trial also reported that erythromycin did not significantly reduce AE in patients without *P. aeruginosa* [ 19]. On the contrary, a more recent study reported that erythromycin could significantly reduce the frequency of AE in patients with *P. aeruginosa* infection who experience one to two exacerbations per year [ 14]. However, there is still no solid, definitive evidence on the relationship between macrolide and the incidence of AE; thus, further research is necessary. In the present study, we retrospectively classified patients with idiopathic bronchiectasis according to the severity of the BRICS and investigated the differences in the effects of macrolides for AE.

HRCT is the gold standard for the definitive diagnosis of bronchiectasis. Previous reports indicated that the severity of HRCT is significantly related to respiratory function and clinical symptoms [ 20- 22]. Consistent with these reports, this study showed that patients in the severe group had more respiratory symptoms and lower respiratory function than patients in the mild and moderate groups (Table **4**). Moreover, the prevalence of *P. aeruginosa* also increased in proportion to the severity, possibly reflecting bacterial changes secondary to repeated infections. The frequency of AE was set as a comparative factor, and significant differences in patients classified by HRCT were confirmed. In this study, no significant difference in the incidence of a single AE was observed between the moderate and severe groups. We hypothesized that the destructive inflammatory cycle may have reduced the therapeutic effectiveness of macrolides. However, a reduction in the incidence of multiple AE was observed regardless of severity, suggesting that continuation of macrolide therapy even after the occurrence of one AE may induce a variety of anti-inflammatory and anti-microbial effects. Also, our research excluded patients with any prior history of AE. We believe that while this selection had little effect on the results of the mild group with a low frequency of AE, it may have influenced the outcome for the incidence of AE in the severity group with a high frequency of AE and various symptoms. On the other hand, the selection of limited patients without a history of AE might indicate the probability of future occurrence of AE in each severity group.

In this study, we adopted the BRICS score, and all patients were classified into three groups accordingly. Regarding the severity scoring system for bronchiectasis, since 1991, some studies have provided their systems, such as the Bhalla score, the modified Reiff score, the bronchiectasis severity index, and the forced expiratory volume in 1 s, age, chronic colonization, extension, and dyspnea (FACED) score [ 15, 23- 25]. The latest BRICS system was categorized only by bronchial dilatation and the number of bronchopulmonary segments with emphysema, and its simplified radiological score was reported to be effective in assessing clinical disease severity in bronchiectasis [ 26]. Through this study, we found it reasonable to use this BRICS system, but close cooperation and discussion between pulmonologists and radiologists were essential regarding the evaluation of HRCT findings.

Nonetheless, this study has some limitations. First, this was a retrospective study, and thus, the type and doses of macrolides were not completely consistent. Also, no patients were treated with azithromycin because its use for bronchiectasis was not permitted in Japan due to health coverage regulations. Second, long-term, low-dose macrolide therapy is also associated with the appearance of macrolide-resistant bacteria, such as drug-resistant oropharyngeal *Streptococci*, but drug-resistance tests were not routinely performed [ 12]. In short, the potential effects of the emergence of drug-resistant bacteria on the therapeutic effect could not be sufficiently ruled out. Third, although the frequency of AE was compared as a marker of therapeutic effect, therapeutic effects for respiratory symptoms and respiratory function were not compared. Additionally, this study was conducted at a single hospital with a limited number of registered patients.

Despite these limitations, we strongly believe that this study has valuable implications for assessing the relationship between long-term, low-dose macrolide therapy and the frequency of AE in idiopathic bronchiectasis classified by severity of the BRICS. Further research regarding the relationship between macrolide therapy and bronchiectasis is recommended.

## CONCLUSION

Long-term, low-dose macrolide therapy might reduce the incidence of multiple AEs, regardless of severity, in patients with idiopathic bronchiectasis without previous AEs. However, macrolide therapy might be less effective in reducing the occurrence of a single AE based on severity. Severity assessment by HRCT might be a useful tool to predict treatment efficacy. Further large-scale clinical trials should be considered to investigate the utility of HRCT in bronchiectasis.

## Figures and Tables

**Table 1:** Severity assessment by the BRICS.

**Score**	**0**	**1**	**2**	**3**
Bronchial dilatation	Absent	Mild (lumen just > diameter of adjacent vessel)	Moderate (lumen 2–3 times > diameter of adjacent vessel)	Sever (lumen > 3 times the diameter of adjacent vessel)
No. of bronchopulmonary segments with emphysema	Absent	1–5	>5	-

**Table 2:** Comparison of characteristics between the groups with and without macrolide therapy at the time of registration.

**Variable**	**With Macrolide**	**Without Macrolide**	**P**
	**(N = 61)**	**(N = 54)**	
Age	71.2 ± 6.9	72.7 ± 7.8	NS
Male	30 (49)	25 (46)	NS
BMI	22.8 ± 2.2	21.2 ± 3.3	NS
Smoking history	-	-	NS
Prior smoker	25 (41)	25 (46)	-
Current smoker	12 (20)	14 (26)	-
Non-smoker	24 (39)	15 (28)	
Symptoms	-	-	-
Cough	45 (74)	45 (83)	NS
Sputum	42 (69)	35 (65)	NS
Bloody sputum	19 (31)	12 (22)	NS
Dyspnea	16 (26)	10 (19)	NS
Bacterial test	-	-	-
* P. aeruginosa*	23 (38)	16 (30)	NS
* H. influenzae*	12 (20)	7 (13)	NS
* S. aureus*	8 (13)	4 (7)	NS
FEV _1_% pred	-	-	-
≥80% pred	10 (16)	6 (11)	NS
≥50–<80 % pred	21 (35)	21 (39)	NS
≥30–<50 % pred	24 (39)	22 (41)	NS
<30% pred	6 (10)	5 (9)	NS
Treatment	-	-	-
ICS/LABA	14 (23)	9 (17)	NS
ICS alone	9 (15)	6 (11)	NS
LABA alone	11 (18)	8 (15)	NS
LAMA	13 (21)	8 (15)	NS
L-carbocisteine	39 (64)	33 (61)	NS
Comorbidities	-	-	-
Asthma	4 (7)	3 (6)	NS
heart disease	7 (11)	4 (7)	NS
CVD	6 (10)	4 (7)	NS
Diabetes mellitus	8 (13)	6 (11)	NS

**Note:** Values are presented as no. (%) or mean ± standard error. BMI, body mass index; CVD, cerebrovascular disease; FEV _1_, forced expiratory volume in 1 second; % pred, percentage of predicted; ICS, inhaled corticosteroid; LABA, long-acting beta-2 agonists; LAMA, long-acting muscarinic antagonists; NS, not significant.

**Table 3:** Comparison of the number of patients with AE between the groups with and without macrolide therapy.

**Variable**	**With** **Macrolide** **(N = 61)**		**Without** ** Macrolide** **(N = 54)**		**OR**	**95% CI**	**P**
	**N**	**%**	**N**	**%**			
**Number of patients with AE**							
Single AE	39	64	29	54	1.52	0.72–3.22	0.34
Multiple AE	8	13	24	44	0.18	0.07–0.47	0.0003

**Note:** Values are presented as no. (%). AE; acute exacerbation; CI, confidence interval; OR, odds ratio.

**Table 4:** Comparison of characteristics between the subgroups with and without macrolide by severity classification at the time of registration.

**-**	**Mild Group**		**-**	**Moderate Group**		**-**	**Severe Group**		**-**
**Variable**	**With**	**Without**	**P**	**With**	**Without**	**P**	**With**	**Without**	**P**
	**(N = 21)**	**(N = 24)**		**(N = 26)**	**(N = 22)**		**(N = 14)**	**(N = 8)**	
Age	71.2 ± 7.1	70.9 ± 8.0	NS	72.7 ± 6.3	72.2 ± 8.2	NS	73.3 ± 8.1	73.1 ± 7.2	NS
Male	11 (52)	13 (54)	NS	13 (50)	---9 (41)	NS	---6 (43)	---3 (8)	NS
BMI	21.7 ± 1.6	21.9 ± 3.0	NS	21.7 ± 2.2	22.2 ± 2.4	NS	20.2 ± 2.8	21.1 ± 2.4	NS
Smoking history	-	-	NS	-	-	NS	-	-	NS
Prior smoker	9 (43)	11 (46)	-	12 (46)	11 (50)	-	4 (29)	---3 (38)	-
Current smoker	5 (24)	7 (29)	-	4 (15)	5 (23)	-	3 (21)	2 (25)	-
Non-smoker	7 (33)	6 (25)		10 (39)	6 (27)		7 (50)	3 (38)	
Symptoms	-	-	-	-	-	-	-	-	-
Cough	11 (52)	17 (71)	NS	20 (77)	20 (91)	NS	14 (100)	8 (100)	NS
Sputum	9 (43)	10 (42)	NS	19 (73)	17 (77)	NS	14 (100)	8 (100)	NS
Bloody sputum	2 (10)	---2 (8)	NS	---9 (35)	---5 (23)	NS	8 (57)	5 (63)	NS
Dyspnea	---1 (5)	---1 (4)	NS	6 (23)	3 (14)	NS	9 (64)	6 (75)	NS
Bacterial test	-	-	-	-	-	-	-	-	-
* P. aeruginosa*	2 (10)	---2 (8)	NS	9 (35)	9 (41)	NS	12 (86)	5 (63)	NS
* H. influenzae*	2 (10)	---1 (4)	NS	4 (15)	---2 (9)	NS	6 (43)	4 (50)	NS
* S. aureus*	---1 (5)	---1 (4)	NS	3 (12)	---1 (5)	NS	4 (29)	2 (25)	NS
FEV _1_% pred	-	-	-	-	-	-	-	-	-
≥80% pred	8 (38)	6 (25)	NS	---2 (8)	---0 (0)	NS	---0 (0)	---0 (0)	NS
≥50–<80 % pred	12 (57)	16 (67)	NS	---6 (23)	4 (18)	NS	3 (21)	1 (12)	NS
≥30–<50 % pred	---1 (5)	---2 (8)	NSNS	17 (65)	16 (73)	NS	6 (43)	4 (50)	NS
<30% pred	---0 (0)	---0 (0)	-	---2 (8)	---2 (9)	NS	4 (29)	3 (38)	NS
Treatment	-	-	-	-	-	-	-	-	-
ICS/LABA	3 (14)	4 (17)	NS	5 (19)	---2 (9)	NS	6 (43)	3 (37)	NS
ICS alone	4 (19)	3 (13)	NS	3 (12)	---1 (5)	NS	2 (14)	2 (25)	NS
LABA alone	2 (10)	4 (17)	NS	4 (15)	---2 (9)	NS	5 (36)	2 (25)	NS
LAMA	3 (14)	---2 (8)	NS	5 (19)	3 (14)	NS	5 (36)	3 (38)	NSNS
L-carbocisteine	4 (19)	10 (42)	NS	21 (81)	15 (68)	NS	14 (100)	8 (100)	-
Comorbidities	-	-	-	-	-	-	-	-	-
Asthma	---1 (5)	---1 (4)	NS	---2 (8)	1 (5)	NS	---1 (7)	1 (12)	NS
Heart disease	---2 (10)	---1 (4)	NS	4 (15)	2 (9)	NS	---1 (7)	1 (12)	NS
CVD	2 (10)	---2 (8)	NS	3 (12)	1 (5)	NS	---1 (7)	1 (12)	NSNS
Diabetes mellitus	2 (10)	---1 (4)	NS	4 (15)	3 (14)	NS	2 (14)	2 (25)	-

**Note:** Values are presented as no. (%) or mean ± standard error. BMI, body mass index; CVD, cerebrovascular disease; FEV _1_, forced expiratory volume in 1 second; % pred, percentage of predicted; ICS, inhaled corticosteroid; LABA, long-acting beta-2 agonists; LAMA, long-acting muscarinic antagonists; NS, not significant.

**Table 5:** Comparison of the number of patients with AE between subgroups with macrolide and without macrolide in mild, moderate, severe groups, respectively.

-	**Mild Group**							**Moderate Group**								**Severe Group**							
	**With Macrolide** (N = 21)		**Without Macrolide** **(N = 24)**		**OR**	**95% CI**	**P**		**With Macrolide** **(N =26)**		**Without Macrolide** **(N = 22)**		**OR**	**95% CI**	**P**		**With Macrolide** **(N = 14)**		**Without Macrolide** **(N = 8)**		**OR**	**95% CI**	**P**
	**N**	**%**	**N**	**%**	-				**N**	**%**	**N**	**%**	-				**N**	**%**	**N**	**%**	-		
**Number of patients with AE**																							
Single	4	19	13	54	0.19	0.05 -0.77	0.029		12	46	10	45	1.02	0.32-3.21	1.00		6	43	2	25	2.25	0.33 -15.3	0.64
Multiple	1	5	8	33	0.10	0.01 -0.88	0.024		4	15	10	45	0.21	0.05-0.84	0.029		3	21	6	75	0.09	0.01 -0.70	0.026

**Note:** Values are presented as no. (%). AE; acute exacerbation; CI, confidence interval; OR, odds ratio.

## Data Availability

The data that support the findings of this study are available on request from the corresponding author [Z.S]. The data are not publicly available due to ethical restrictions.

## LIST OF ABBREVIATIONS

HRCT =: High-resolution computed tomography
AE =: Acute exacerbation
BRICS =: Bronchiectasis radiologically indexed CT score
